# Efficient synthesis of triarylamine-based dyes for *p*-type dye-sensitized solar cells

**DOI:** 10.1038/srep26263

**Published:** 2016-05-19

**Authors:** Martin Wild, Jan Griebel, Anna Hajduk, Dirk Friedrich, Annegret Stark, Bernd Abel, Katrin R. Siefermann

**Affiliations:** 1 Department of Chemistry, Leibniz Institute of Surface Modification (IOM), Permoserstraße 15, 04318 Leipzig, Germany; 2 Wilhelm-Ostwald-Institute for Physical and Theoretical Chemistry, University Leipzig, Linnéstraße 2, 04103 Leipzig, Germany; 3 SMRI Sugarcane Biorefinery Research Chair, University of KwaZulu-Natal, College of Agriculture, Engineering and Science School of Engineering, Howard College Campus, Durban, South Africa

## Abstract

The class of triarylamine-based dyes has proven great potential as efficient light absorbers in inverse (*p*-type) dye sensitized solar cells (DSSCs). However, detailed investigation and further improvement of *p*-type DSSCs is strongly hindered by the fact that available synthesis routes of triarylamine-based dyes are inefficient and particularly demanding with regard to time and costs. Here, we report on an efficient synthesis strategy for triarylamine-based dyes for *p*-type DSSCs. A protocol for the synthesis of the dye-precursor (4-(bis(4-bromophenyl)amino)benzoic acid) is presented along with its X-ray crystal structure. The dye precursor is obtained from the commercially available 4(diphenylamino)benzaldehyde in a yield of 87% and serves as a starting point for the synthesis of various triarylamine-based dyes. Starting from the precursor we further describe a synthesis protocol for the dye 4-{bis[4′-(2,2-dicyanovinyl)-[1,1′-biphenyl]-4-yl]amino}benzoic acid (also known as dye P4) in a yield of 74%. All synthesis steps are characterized by high yields and high purities without the need for laborious purification steps and thus fulfill essential requirements for scale-up.

The transition from a fossil fuel-powered to a sustainable world economy is one of the major challenges facing the energy industry today and in the future. Among the available sources of renewable energy, solar photovoltaics is considered to have the highest potential to cover a major share of the future energy needs. In order to exploit this potential, a safe, efficient, environmentally friendly and cost-effective method of harvesting solar energy is required. Dye-sensitized solar cells (DSSCs) belong to the third generation solar cell technologies and have the potential to fulfill these requirements^1^. The essential step in the development of DSSCs was made by the Grätzel group in 1991^2^. Until today, a combination of basic and applied research efforts on these devices has resulted in solar-to-electric power conversion efficiencies (PCE) exceeding 12% for *n*-type DSSCs^3^,^4^,^5^,^6^,^7^. In *n*-type DSSCs, the initial step is the electronic excitation of the dye by light, and the subsequent injection of the excited electron into the semiconductor.

Over the past years, “inverse” DSSCs of the *p*-type have received increasing attention. In *p*-type cells, the initial step is the light-induced excitation of an electron from the HOMO to the LUMO orbital of the dye. Subsequently, the hole created in the HOMO is filled by an electron from the *p*-type semiconductor (e.g. nickel oxide). This is followed by the transfer of the excited electron from the LUMO to the redox couple (e.g. I^−^/I_3_^−^).

*p*-type DSSCs are of particular interest for tandem devices, in which a dye-sensitized photoanode (of the *n*-type) is connected in series with a dye-sensitized photocathode (of the *p*-type)^8^,^9^,^10^,^11^. The theoretical efficiency of such a tandem cell may be as high as PCE = 43%^10^.

However, the development of these tandem devices is hindered by the low performance of *p*-type DSSCs. Currently, the highest PCE reported for a *p*-type DSSC is less than 0.5%^8^,^12^,^13^,^14^,^15^, and thus far below the PCE values of ~12% obtained for *n*-type DSSCs^3^. This low value can be partly attributed to the limited understanding of *p*-type DSSC devices. While many different dyes have been tested with regard to their performance in *p*-type DSSCs^9^,^12^,^16^,^17^,^18^,^19^,^20^,^21^,^22^,^23^, only few systematic and mechanistic studies are available to date^8^,^10^,^18^,^19^,^24^,^25^,^26^,^27^,^28^,^29^,^30^,^31^,^32^,^33^,^34^. The (counterpart) *n*-type DSSC is an outstanding example for how systematic materials studies^3^,^4^,^35^ and an increasing mechanistic understanding^36^,^37^,^38^ have led to a successive improvement of efficiencies. Besides, design concepts which have been successfully applied in *n*-type DSSCs should be transferred to *p*-type DSSCs in order to improve their efficiency. A promising direction is to transfer novel counter electrode designs from *n*-type DSSCs to *p*-type DSSCs^39^. For *n*-type DSSCs it has recently been shown that counter electrodes based on metal alloys^40^,^41^,^42^ and novel nanostructured polymers^43^ exhibit superior properties with regard to electrode stability and solar cell efficiency when compared to conventional platinum counter electrodes. Another promising approach for improving *p*-type DSSC efficiencies is to include tailored quantum dot antennas into the DSSC, in order to enhance light absorption and broadening the absorption spectrum^44^. In this context, novel and less toxic types of quantum dots are of particular interest^45^,^46^. However, all these promising further investigations on *p*-type DSSCs require the materials to be available in sufficient amounts. Especially for triarylamine-based dyes, which are up to now the best-performing dyes for *p*-type DSSCs and further show excellent long term stabilities comparable to ruthenium based dyes^47^, this poses a challenge due to the time-demanding and inefficient nature of the available synthesis routes^10^,^23^,^25^,^26^,^27^. Figure 1b gives an overview of various triarylamine-based dyes for DSSCs. The general structure is the triarylamine building block with one carboxylic acid moiety, which serves as the anchor group to the *p*-type semiconductor. Figure 1 also summarizes synthesis yields for the dyes as reported in the literature. These synthesis yields range from 6% to 24%, using the commercially available 4-(diphenylamino)benzaldehyde as the starting compound in all cases. Here, we present an efficient two-step synthesis route from this starting compound to the substance (4-(bis(4-bromophenyl)amino)benzoic acid) (yield = 87%). This substance is an ideal precursor for the synthesis of a broad variety of triarylamine-based dyes via cross coupling reactions (Fig. 1)^10^,^24^,^25^,^27^. Furthermore, we exemplary describe the further conversion of the precursor into the dye 4-{bis[4′-(2,2-dicyanovinyl)-[1,1′-biphenyl]-4-yl]amino}benzoic acid. In the literature, this compound is known as dye P4 and an overall solar cell efficiency of 0.09% has been reported for a DSSC with P4 on the *p*-type semiconductor NiO^25^. Our efficient 2-step synthesis route from the precursor to dye P4 features a yield of 74%. With this, the overall synthesis protocol from the starting compound to dye P4 offers a yield of 64%. This value is significantly higher than previously reported yields for dye P4 and related dyes (see Fig. 1b). Our optimized synthesis protocol further delivers high purity in every synthesis step without the need for elaborate and costly purification steps, such as column chromatography. Starting from the dye precursor, the protocol can easily be adapted for conversion into other dyes, where a similarly high yield can be expected.

## Results and Discussion

Figure 2 illustrates the two-step synthesis from the starting compound to the dye precursor. At first, a bromination of 4(diphenylamino)benzaldehyde yields 4-(bis(4-bromophenyl)amino)benzaldehyde (**1**), followed by an oxidation of **1** to 4-(bis(4-bromophenyl)amino)benzoic acid (**2**). A Suzuki cross coupling of **2** with 4-formylphenylboronic acid yields 4-(bis(4′-formylbiphenyl-4-yl)amino)benzoic acid (**3**) and finally a Knoevenagel condensation of **3** and malonodinitrile gives the desired dye 4-{bis[4′-(2,2-dicyanovinyl)-[1,1′-biphenyl]-4-yl]amino}benzoic acid (**dye P4**).

### Synthesis of 4-(bis(4-bromophenyl)amino)benzaldehyde (1)

We have systematically optimized the synthesis protocol with regard to the addition time and the concentration of the added bromine. The highest yield of 96% was achieved when slowly adding a dilute solution of bromine in dichloromethane (about 0.5 M) to the 4-(diphenylamino)benzaldehyde solution at 0 °C. The bromine solution was added within 3.5 h, followed by 3 h stirring at room temperature, 16 h stirring at 30 °C and 3 h stirring under reflux (Fig. 2, i). With this procedure, product **1** was obtained in such a high purity that no further purification was necessary. The bromination of 4-(diphenylamino)benzaldehyde has previously been described by Qin *et al*.^24^ with a yield of 63% and Xu *et al*.^48^ with a yield of 80%. In both cases, column chromatography was required to obtain the product in sufficient purity. In contrast to our protocol, they carried out the bromination via the addition of pure bromine over a shorter period of time. According to our findings, the concentration of the bromine and the time over which it is added have a strong effect on the yield and the purity of **1**. Evidently, the low bromine concentration and its slow addition suppresses the formation of unwanted multiple substituted products and makes purification via column chromatography unnecessary.

### Synthesis of 4-(bis(4-bromophenyl)amino)benzoic acid (2)

We find that the dye precursor **2** is obtained in a yield of 90% with the following procedure: **1** was dissolved in toluene and added slowly to the suspension of silver(I)oxide (4 equiv.) and sodium hydroxide (8 equiv.) in ethanol. After 3 h, the reaction was complete (determined by thin layer chromatography). After another 90 min of stirring at room temperature, the mixture was decanted and precooled HCl (10 wt%) was added. An extraction with ethyl acetate yields **2** in high purity and with a yield of 90%, hence not requiring any further purification.

Compared to the procedure reported by Qin *et al*.^24^ with a yield of 78% (after column chromatography), we were able to decrease the amount of required silver(I)oxide (from 10 equiv. to 4 equiv.) as well as sodium hydroxide (from 55 equiv. to 8 equiv.). This finding indicates that the lower hydroxide concentration leads to a higher oxidation potential of the couple Ag^+^/Ag, as there are more protons available in this case (see equations (1) to (3)).


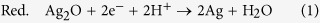



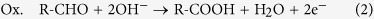



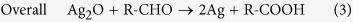


As opposed to our two-step synthesis of the dye precursor, Lefebvre *et al*.^10^ use *N*-bromosuccinimide as brominating agent in dry tetrahydrofuran under an atmosphere of argon, and use the residue of the bromination reaction without purification for the oxidation. The oxidation of **1** to **2** was carried out in a refluxing acetone/water mixture with potassium permanganate and the residue was purified by column chromatography using a gradient of acetic acid and chloroform. The overall yield of the two reactions was 88% (1.88 g)^10^. This yield is comparable to our yield of the two reaction steps (87%). However, an advantage of our procedure is that it does not require work under inert conditions. Furthermore, the second reaction step of the synthesis of Lefebvre *et al*. requires column chromatography with a solvent gradient, which makes the overall procedure more demanding^10^.

We further performed an X-ray single crystal analysis on crystals of the dye precursor (Crystallographic data (excluding structure factors) for the structures in this paper have been deposited with the Cambridge Crystallographic Data Centre as supplementary publication CCDC 1001466. Copies of the data can be obtained, free of charge, on application to CCDC, 12 Union Road, Cambridge CB2 1EZ, UK, (fax: +44-(0)1223–336033 or e-mail: deposit@ccdc.cam.ac.uk)). The compound (**2**) crystallizes in the monoclinic space group P 2_1_/c with eight formula units per unit cell. The asymmetric unit consists of two molecules (Fig. 3). Each molecule forms hydrogen bonds with one neighboring molecule via the carbonic acid moieties. Two different hydrogen bond lengths between acidic proton and carbonyl oxygen atoms of neighboring molecules can be distinguished, 179.9 (3) pm and 182.5 (3) pm. The bonding geometry of the nitrogen atoms is trigonal planar with out-of-plane distances below 10 pm. The distance between the nitrogen atoms and the binding carbon atoms of the phenyl carboxylic acid is around 140 pm. It is slightly shorter than the N-C-distance to the phenyl bromide groups, which is around 143 pm. A summary of all crystallographic data is given in the supporting information S5.

We further determined the extinction coefficient of the dye precursor in acetonitrile: ε_314 nm, acetonitrile_ = 25200 l mol^−1^ cm^−1^. The UV/Vis absorption spectrum is presented in the supporting information S11.

### Synthesis of 4-(bis(4′-formylbiphenyl-4-yl)amino)benzoic acid (3)

**Figure 1:**
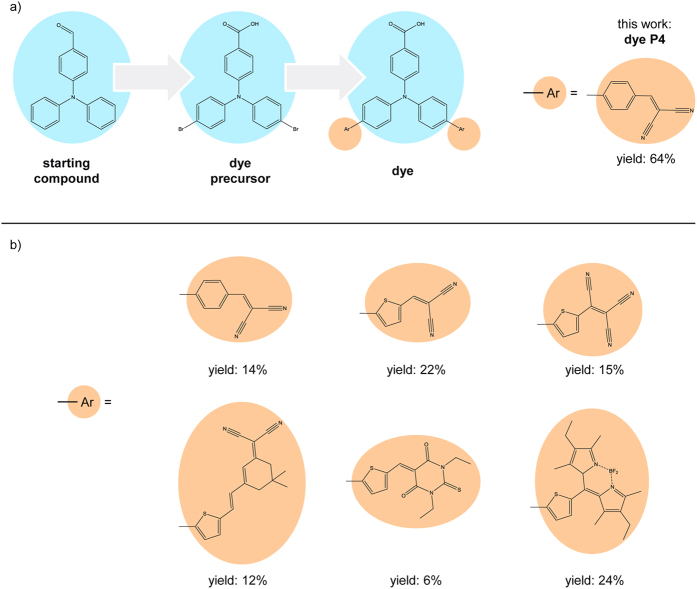
(**a**) General synthesis route for a variety of triarylamine-based dyes. This work presents a facile and efficient protocol for the synthesis of dye P4 in an overall yield of 64%. (**b**) Overview of triarylamine-based dyes for *p*-type DSSCs and synthesis yields as reported in the literature^10^,^25^,^26^,^27^. Synthesis yields all refer to the starting compound shown in (**a**).

**Figure 2:**
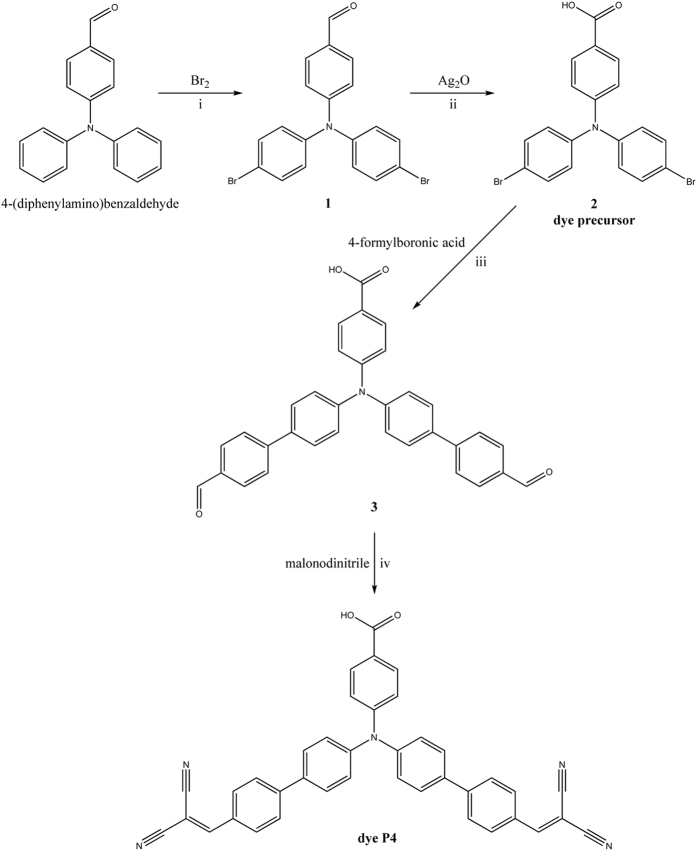
Synthesis of dye P4. i: a) Br_2_ in CH_2_Cl_2_, cooling, b) room temperature (rt), 3 h, c) 30 °C, 16 h d) reflux, 3 h, e) KOH_aq._; ii: a) silver(I)oxide (4 equiv.), NaOH (8 equiv.), ethanol, toluene, b) rt, 4 h, c) HCl_aq_.; iii: a) Pd(PPh_3_)_4_, 4-formylphenylboronic acid, K_2_CO_3_, dimethylacetamide, water b) reflux, 24 h; iv: a) malonodinitrile (3.5 equiv.), ammonium acetate (2.5 equiv.), acetic acid (2 equiv.), toluene b) reflux, 30 h.

**Figure 3:**
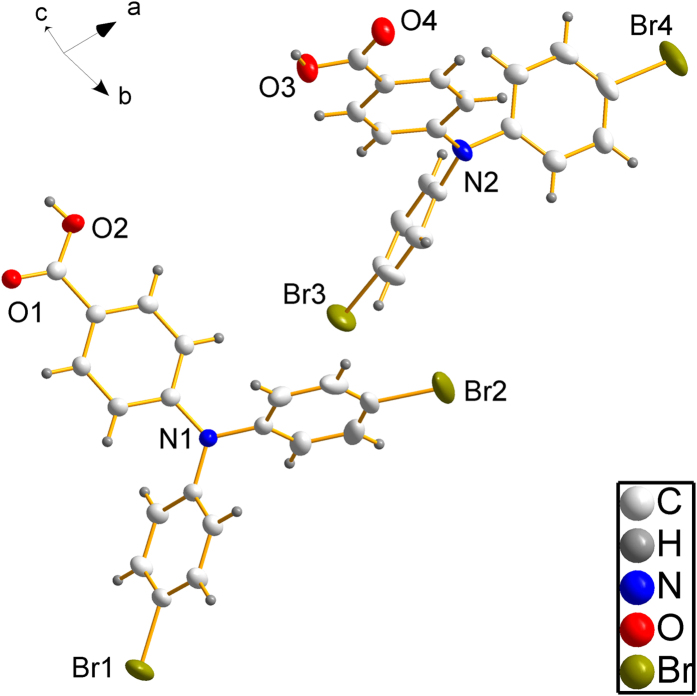
Asymmetric unit of the dye precursor (2) containing two molecules; non-hydrogen atoms are displayed as ellipsoids with a probability of 50%.

The third reaction step in the synthesis of the triarylamine-based dyes in Fig. 1 is a Suzuki coupling of the dye precursor **2** with the appropriate boronic acid. In general, Suzuki couplings can be performed with a plethora of reagents, allowing for the facile synthesis and investigation of series of homologues in a library-type approach. In the dye synthesis, the Suzuki coupling is the essential step for the preparation of different dyes. In the following, we exemplary present an efficient synthesis protocol for **dye P4**. We perform the Suzuki coupling reaction with the catalyst tetrakis(triphenylphosphine)palladium(0) (Pd(PPh_3_)_4_). The dye precursor **2** was mixed with 4-formylphenylboronic acid, potassium carbonate, dimethylacetamide, and water and heated under reflux for 24 hours. The product **3** is purified via recrystallization and obtained in a yield of 80% (0.181 g).

The Suzuki coupling reaction of **2** with formylphenylboronic acid has previously been described with the catalyst [1,1′-bis(diphenylphosphino)ferrocene]dichloropalladium(II) (complex with dichloromethane)^25^. The respective reaction was carried out in a microwave oven and delivered a yield of 59% after purification via column chromatography^25^. The optimized protocol presented here allows to obtain a higher yield, while a factor of 3 less catalyst material is required (0.012 mmol Pd^2+^ per 100 mg product, compared to 0.038 mmol Pd^2+^ per 100 mg product in ref. 25) and no column chromatography is necessary.

### Synthesis of 4-{bis[4′-(2,2-dicyanovinyl)-[1,1′-biphenyl]-4-yl]amino}benzoic acid (dye P4)

The last reaction step in the synthesis of **dye P4** is a Knoevenagel condensation using the dialdehyde **3** and malonodinitrile. We find that the Knoevenagel variant of Cope^49^ including ammonium acetate as a base delivers the product in a high yield. The reaction of **3** with malonodinitrile, ammonium acetate and acetic acid in refluxing toluene yields **dye P4** in a yield of 92% and without the need for any further purification. For comparison, a protocol reported by Qin *et al*.^25^ delivers a yield of 60% (after column chromatography).

The purity of our final product **P4** is 96% according to ^1^H-NMR and is thus comparable to the purity of commercially available dyes for DSSCs (typically 95%, NMR).

The UV/Vis absorption spectrum of dye P4 in acetonitrile is presented in the supporting information S13 and shows two absorption bands at 326 nm and 419 nm with extinction coefficients of ε_326 nm, acetonitrile_ = 42400 l mol^−1^ cm^−1^ and ε_419 nm, acetonitrile_ = 39200 l mol^−1^ cm^−1^, respectively. Values are in agreement with those reported in the literature^25^.

The development of *p*-type dye-sensitized solar cells is strongly hindered by the fact that the necessary dyes are not readily available. Here, we reported on a facile, efficient and scalable synthesis strategy for triarylamine-based dyes for *p*-type DSSCs. All presented reaction steps feature high yields (>80%) and deliver the product in a high purity so that no laborious purification steps (such as column chromatography) are necessary. Starting from a commercially available compound, a two-step synthesis protocol is presented, which delivers a dye precursor in a yield of 87%. This dye precursor is the starting point for the synthesis of various triarylamine-based dyes. We exemplary present a two-step protocol for the transformation of the dye precursor into the dye (4-{bis[4′-(2,2-dicyanovinyl)-[1,1′-biphenyl]-4-yl]amino}benzoic acid), also known as dye P4. The overall yield for this dye is 64% and thus significantly higher than previously reported yields for triarylamine-based dyes, which range from 6% to 24%^10^,^25^,^26^,^27^. This synthesis protocol can easily be adapted for the conversion of the dye precursor into other dyes, where a similarly high yield can be expected. With this, triarylamine-based dyes are now accessible for many detailed and systematic studies on *p*-type DSSCs and beyond. Besides, the presented synthesis route is optimized with regard to yield, chemicals, time, and cost and may thus even provide a starting point for production of dyes beyond the laboratory scale.

## Methods

### Materials and Characterization

Chemicals and solvents were purchased from VWR and Sigma Aldrich in HPLC grade and were used as received. The products were characterized by ^1^H-NMR and ^13^C-NMR using a *Bruker Avance Ultra Shield 600* *MHz* spectrometer with a 5 mm BBO probe head. The chemical shifts (δ) are given in parts per million (ppm) and the reference signal was the solvent signal. MS spectra were recorded on a *Bruker Daltonics esquire 3000plus* equipped with an ion trap and electrospray ionization. The sample was injected via a syringe pump with a constant flow rate of 240 μl h^−1^. Nitrogen was used as spray and dry gas and was heated to 300 °C. The MS spectra are given in m/z ratio. The UV/Vis spectra were recorded on a *SHIMADZU UV-2101PC* spectrometer using a 10 mm cuvette. IR measurements were carried out on a *FTS 6000* spectrometer from BIO-RAD using the ATR modus. The spectrometer was equipped with a Golden Gate ATR accessory from Specac with a diamond crystal. For thin layer chromatography, TLC Silica gel 60 F254 plates from Merck were used. The crystal structure was determined on a *STOE IPDS-2T* diffractometer. Crystal structures were determined by measurements with an area detector system from STOE (IPDS 1 or IPDS-2T). The single crystals were covered with mineral oil and mounted on a thin glass fiber attached to the goniometer head. The prepared single crystal samples were cooled down to 213 K (IPDS 1) or 180 K or 100 K (IPDS-2T) before measurement, thus freezing the mineral oil and fixing the crystal. Source of radiation was a sealed X-ray tube with a Mo-anode (l (Mo-Ka) = 71.073 pm, 50 kV, 40 mA) and a graphite monochromator. The data collection, unit cell determination, integration and absorption correction were handled using the Program X-Area. The crystal structures were solved by direct methods using the interface program WINGX^50^ including the programs SHELXS-97^51^ and SIR-92^52^ for crystal structure determination. Refinement was performed with the program SHELXL-97^51^. Figures of structures were created using the program DIAMOND 3. Files (.cif, .fcf, .hkl) of single crystal structures presented in this publication are deposited in the Cambridge Structural Database.

### Synthesis of 4-(bis(4-bromophenyl)amino)benzaldehyde (1)

In a two-neck round bottom flask (500 ml), equipped with a reflux condenser and a dropping funnel, 3.057 g (11.184 mmol) of 4-(diphenylamino)benzaldehyde were dissolved in 100 ml dichloromethane and stirred while cooling with an ice bath. Simultaneously, 4.336 g (27.133 mmol) of bromine were dissolved in 50 ml dichloromethane. After cooling the 4-(diphenylamino)benzaldehyde solution to less than 5 °C, 42 ml (3.642 g, 22.791 mmol) of the bromine solution were added slowly over a time of 210 min. During the addition of the bromine solution, the color of the reaction solution changed from yellow/orange to brown. After the addition, the reaction mixture was stirred at room temperature for 180 min followed by stirring for 16 h at 30 °C and further stirring for 180 min under reflux. Afterwards, an aqueous solution of potassium hydroxide (1.516 g (27.018 mmol) in 150 ml deionized water) was added and a color change from brown to yellowish green was observed. The aqueous phase was extracted with dichloromethane (3 × 50 ml) and the combined organic phases were extracted with deionized water (2 × 100 ml). The organic layer was dried over anhydrous sodium sulfate. After filtration, the solvent was removed by rotary evaporation to give the yellow greenish product in quantitative yield: 4.629 g, 96.0% (>97% purity according to ^1^H-NMR spectrum). Petroleum ether/dichloromethane, v/v = 1/2, R_f_ = 0.64. ^1^H-NMR (600 MHz, acetone-d_6_): [ppm] = 9.87 (1 H, s), 7.79 (2 H, dt, *J*_*t*_ = 2.35 Hz, *J*_*d*_ = 9.08 Hz), 7.56 (4 H, dt, *J*_*t*_ = 2.64 Hz, *J*_*d*_ = 9,48 Hz), 7.15 (4 H, dt, *J*_*t*_ = 2.64 Hz, *J*_*d*_ = 9.42 Hz), 7.09 (2 H, dt, *J*_*t*_ = 2.28 Hz, *J*_*d*_ = 9.12 Hz). ^13^C-NMR (151 MHz, acetone-d_6_): [ppm] = 191.36 (1 C, s), 153.71 (1 C, s), 146.87 (2 C, s), 134.32 (4 C, s), 132.48 (2 C, s), 132.01 (1 C, s), 129.23 (4 C, s), 121.86 (2 C, s), 118.92 (2 C, s). MS (ESI) [m/z] = 453.7 (100%) [M+Na]^+^.

### Synthesis of 4-(bis(4-bromophenyl)amino)benzoic acid (2)

In a two-neck round bottom flask (250 ml), connected to a reflux condenser, 2.500 g (10.787 mmol) silver(I)oxide were suspended in 100 ml ethanol and 0.838 g (20.949 mmol) sodium hydroxide were added stepwise. To this suspension, a solution of 1.011 g (2.344 mmol) **1** in 15 ml toluene was added within 30 min. The reaction mixture was stirred at room temperature for 4 h. The mixture was decanted into 100 ml precooled hydrochloric acid (10 wt%). The silver oxide residue was extracted with ethanol (2 × 30 ml) to transfer the product quantitatively. The volume of the resulting solution was reduced using the rotary evaporator until a precipitation was observed. Subsequently, 100 ml deionized water and 300 ml ethyl acetate (to dissolve the precipitate) was added and the aqueous phase was extracted with ethyl acetate (2 × 100 ml). The combined organic phases were extracted with deionized water (2 × 100 ml) and the resulting organic phase was dried over anhydrous sodium sulfate. After filtration, the solvent was removed using a rotary evaporator to give the beige-colored crystalline product: 0.948 g, 90.4% (>95% purity according to ^1^H-NMR spectrum). *n*-Hexane/ethyl acetate, v/v = 2/1, R_f_ = 0.55. ^1^H-NMR (600 MHz, acetone-d_6_): [ppm] = 11.05 (1 H, s), 7.93 (2 H, dt, *J*_*t*_ = 2.31 Hz, *J*_*d*_ = 9.12 Hz), 7.53 (4 H, dt, *J*_*t*_ = 2.64 Hz, *J*_*d*_ = 9.48 Hz), 7.11 (4 H, dt, *J*_*t*_ = 2.64 Hz, *J*_*d*_ = 9.48 Hz), 7.06 (2 H, dt, *J*_*t*_ = 2.32 Hz, *J*_*d*_ = 9.22 Hz). ^13^C-NMR (151 MHz, acetone-d_6_): [ppm] = 167.16 (1 C, s), 152.07 (1 C, s), 146.82 (2 C, s), 133.72 (4 C, s), 132.13 (2 C, s), 128.24 (4 C, s), 124.83 (1 C, s), 121.96 (2 C, s), 117.77 (2 C, s). MS (ESI) [m/z] = 445.8 (100%) [M-H]^−^. IR [cm^−1^] = 2741 (w), 1666 (s), 1580 (s), 1485 (s), 1283 (s), 660 (w). ε_313.5, acetonitrile_ = 25200 l mol^−1^ cm^−1^.

### Synthesis of 4-(bis(4′-formylbiphenyl-4-yl)amino)benzoic acid (3)

0.203 g (0.454 mmol) **2**, 0.032 g (0.028 mmol) Pd(PPh_3_)_4_, 0.270 g (1.801 mmol) 4-formylphenylboronic acid, and 0.498 g (3.603 mmol) potassium carbonate were dissolved in 31 ml dimethylacetamide and 9 ml water. The mixture was heated under reflux for 24 h. After cooling to room temperature, the mixture was poured into 100 ml of a saturated aqueous solution of ammonium chloride. The resulting aqueous phase was extracted with dichloromethane (3 × 50 ml). The combined organic phases were extracted with brine (2 × 100 ml) and dried over anhydrous sodium sulfate. After filtration, the solvent was removed using a rotary evaporator. The residue was dissolved in 15 ml dichloromethane and 5 ml toluene were added. After removing dichloromethane using a rotary evaporator, the mixture was stored in a freezer (−20 °C) to complete crystallization. The precipitate was filtered, washed with precooled toluene (2 × 5 ml) and dried under vacuum to give a yellow product: 0.181 g, 80.2% (95% purity according to ^1^H-NMR spectrum). Acetone/petroleum ether, v/v = 2/3, R_f_ = 0.58. ^1^H-NMR (600 MHz, acetone-d_6_): [ppm] = 11.04 (1 H, s), 10.09 (2 H, s), 8.02 (4 H, d, *J*_*d*_ = 8.34 Hz), 7.98 (2 H, d, *J*_*d*_ = 8.76 Hz), 7.94 (4 H, d, *J*_*d*_ = 8.22 Hz), 7.83 (4 H, d, *J*_*d*_ = 8.58 Hz), 7.33 (4 H, d, *J*_*d*_ = 8.58 Hz), 7.18 (2 H, d, *J*_*d*_ = 8.70 Hz). ^13^C-NMR (151 MHz, acetone-d_6_): [ppm] = 191.18 (2 C, s), 167.85 (1 C, s), 150.92 (1 C, s), 146.65 (2 C, s), 145.31 (2 C, s), 135.14 (2 C, s), 134.87 (2 C, s), 134.18 (1 C, s), 130.79 (2 C, s), 129.71 (4 C, s), 128.18 (4 C, s), 126.71 (4 C, s), 125.32 (4 C), 121.20 (2 C, s). MS (ESI) [m/z] = 496.0 (100%) [M-H]^−^. IR [cm^−1^] = 2741 (w), 1670 (s), 1582 (s), 1487 (s), 1283 (s).

### Synthesis of 4-{bis[4′-(2,2-dicyanovinyl)-[1,1′-biphenyl]-4-yl]amino}benzoic acid (dye P4)

In a round bottom flask (50 ml), 0.110 g (0.221 mmol) **3**, 0.050 g (0.757 mmol) malonodinitrile and 0.046 g (0.597 mmol) ammonium acetate were dissolved in 15 ml toluene and to the resulting solution 0.028 g (0.460 mmol) glacial acetic acid were added. The mixture was heated under reflux for 30 h. The progress of the reaction was followed by ^1^H-NMR (ratio between **3** and **dye P4**). After 30 h reflux, no starting material (**3**) was detectable any more, indicating completion of the reaction. For purification, the boiling reaction mixture was filtered by pouring it into a frit. The flask was rinsed two times with boiling toluene (10 ml each), which was then poured over the residue in the frit and thus added to the filtrate. Additionally, the residue in the frit was extracted with boiling toluene (3 × 10 ml). The filtrate was cooled to room temperature, slightly concentrated using a rotary evaporator, and stored in a freezer. Dye P4 precipitates from the filtrate as a red solid: 0.121  g, 92.2% (96% purity according to ^1^H-NMR spectrum). ^1^H-NMR (600 MHz, acetone-d_6_): [ppm] = 11.06 (1 H, s), 8.35 (2 H, s), 8.15 (4 H, d, *J*_*d*_ = 8.40), 8.00 (6 H, m), 7.88 (4 H, d, *J*_*d*_ = 8.64), 7.34 (4 H, d, *J*_*d*_ = 8.64), 7.21 (2 H, d, *J*_*d*_ = 8.70). MS (ESI) m/z: 592.2 (100%) [M-H]^−^. IR [cm^−1^] = 2741 (w), 2224 (w), 1674 (s), 1574 (s), 1487 (s), 1281 (s). ε_325.5, acetonitrile_ = 39200 l mol^−1^ cm^−1^, ε_418.5, acetonitrile_ = 42400 l mol^−1^ cm^−1^.

## Additional Information

**How to cite this article**: Wild, M. *et al*. Efficient synthesis of triarylamine-based dyes for *p*-type dye-sensitized solar cells. *Sci. Rep*. **6**, 26263; doi: 10.1038/srep26263 (2016).

## Supplementary Material

Supplementary Information
