# Supplementary material for: Efficient synthesis of triarylamine-based dyes for p-type dye-sensitized solar cells
Source: Sci Rep. 2016 May 19;6:26263. doi: 10.1038/srep26263 (PMC4872536; doi:10.1038/srep26263)
Supplement: Supplementary Information [file srep26263-s1.pdf]

Efficient synthesis of triarylamine-based dyes for *p*-type dye-sensitized solar cells

Martin Wild[a,b], Jan Griebel[a], Anna Hajduk[b], Dirk Friedrich[b], Annegret Stark[c], Bernd Abel[a,b], Katrin R. Siefermann[a,*]

[a] *Leibniz Institute of Surface Modification, Chemical Department, Permoserstraße 15, 04318 Leipzig, Germany.*

[b] *Wilhelm-Ostwald-Institute for Physical and Theoretical Chemistry, University Leipzig, Linnéstraße 2, 04103 Leipzig, Germany.*

[c] *SMRI Sugarcane Biorefinery Research Chair, University of KwaZulu-Natal, College of Agriculture, Engineering and Science School of Engineering, Howard College Campus, Durban, South Africa.*

Contents

S1 NMR spectra

S2 IR spectra

S3 UV/Vis spectra

S4 MS spectra

S5 X-Ray crystallographic details

[*] *Corresponding author: Tel.: +49 (0) 341 235 3364; fax: +49 (0) 341 235 2584; E-mail address: katrin.siefermann@iom-leipzig.de (K. R. Siefermann).*

S1 NMR spectra

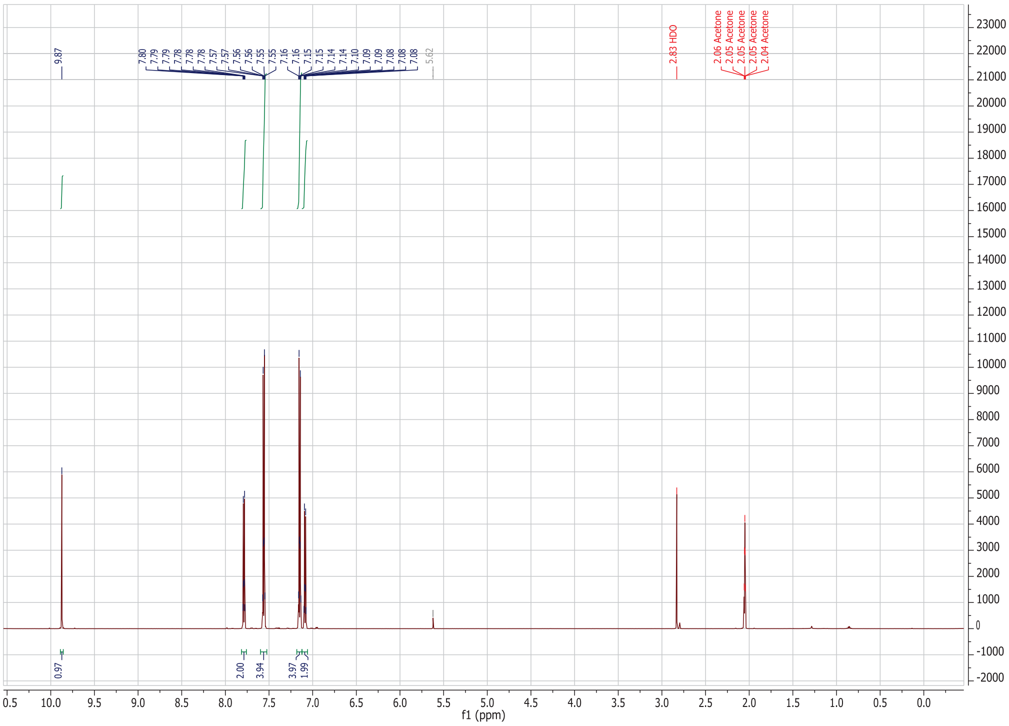

Figure S1 ^{1}H-NMR spectrum of compound **1** (4-(bis(4-bromophenyl)amino)benzaldehyde) (600 MHz, acetone-d_6).

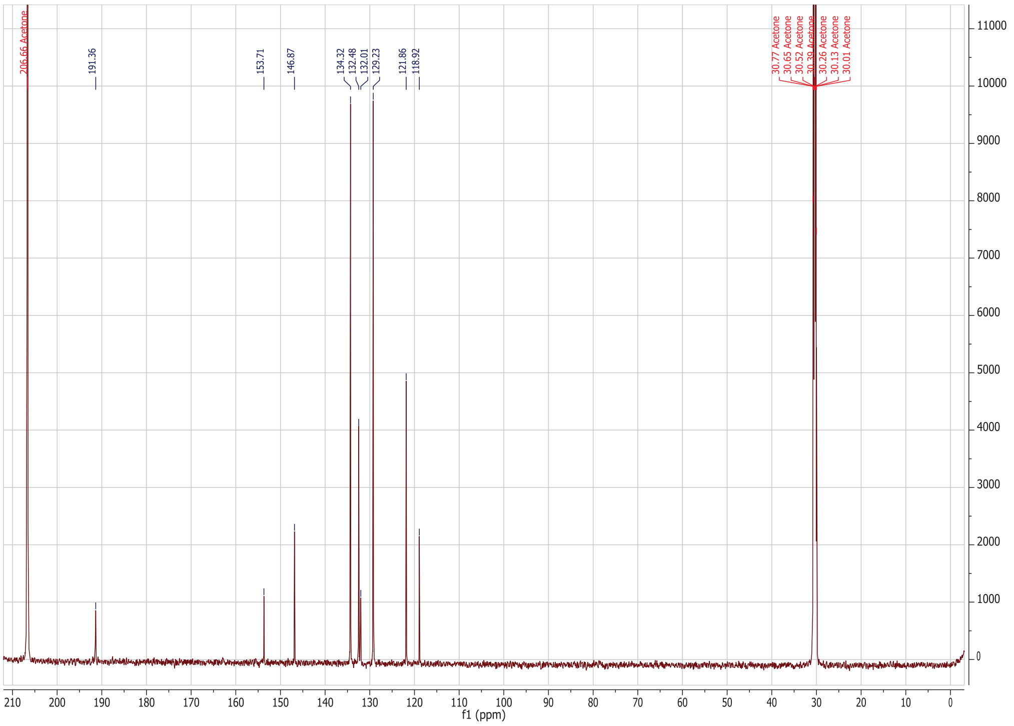

Figure S2 ^{13}C-NMR spectrum of compound **1** (4-(bis(4-bromophenyl)amino)benzaldehyde) (151 MHz, acetone-d_6).

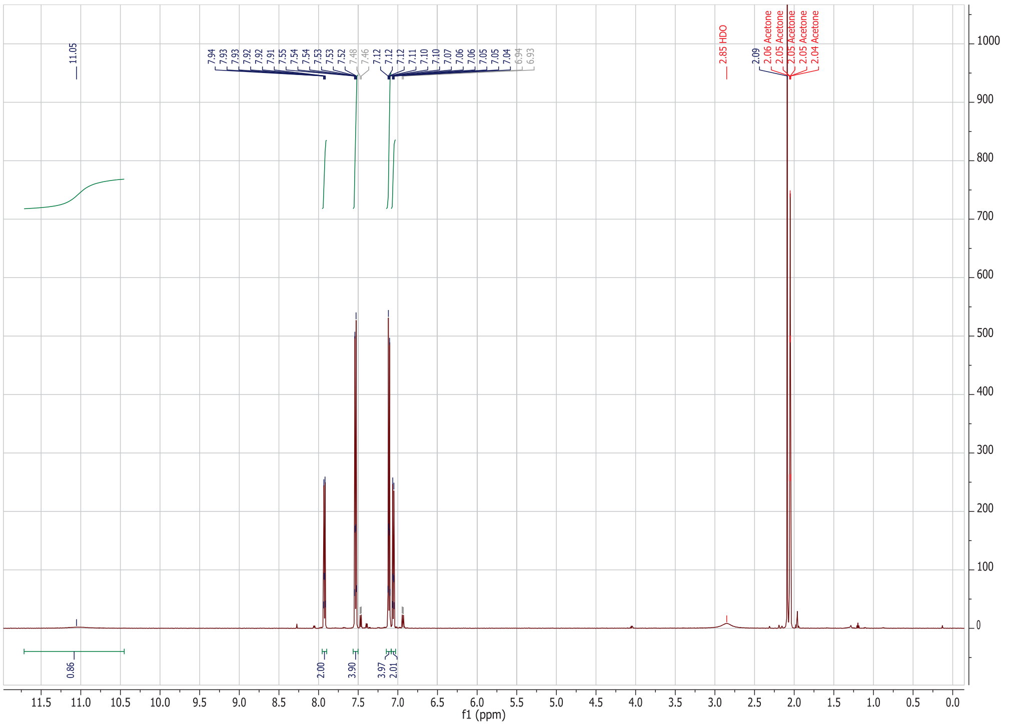

Figure S3 ^{1}H-NMR spectrum of compound **2** (4-(bis(4-bromophenyl)amino)benzoic acid, dye precursor) (600 MHz, acetone-d_6).

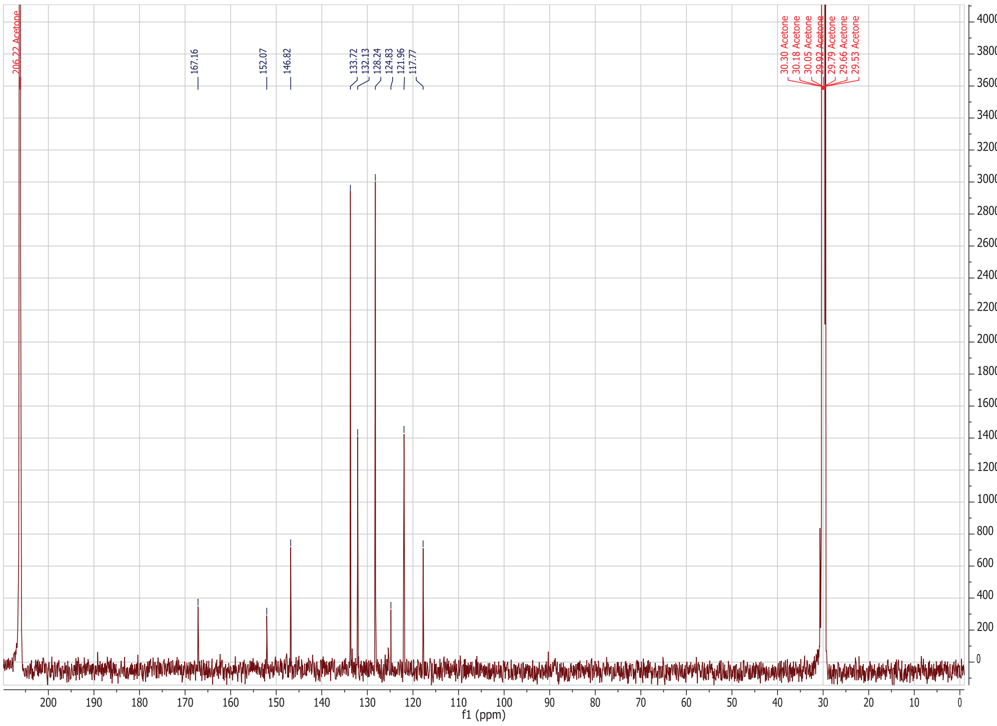

Figure S4 ^{13}C-NMR spectrum of compound **2** (4-(bis(4-bromophenyl)amino)benzoic acid, dye precursor) (151 MHz, acetone-d_6).

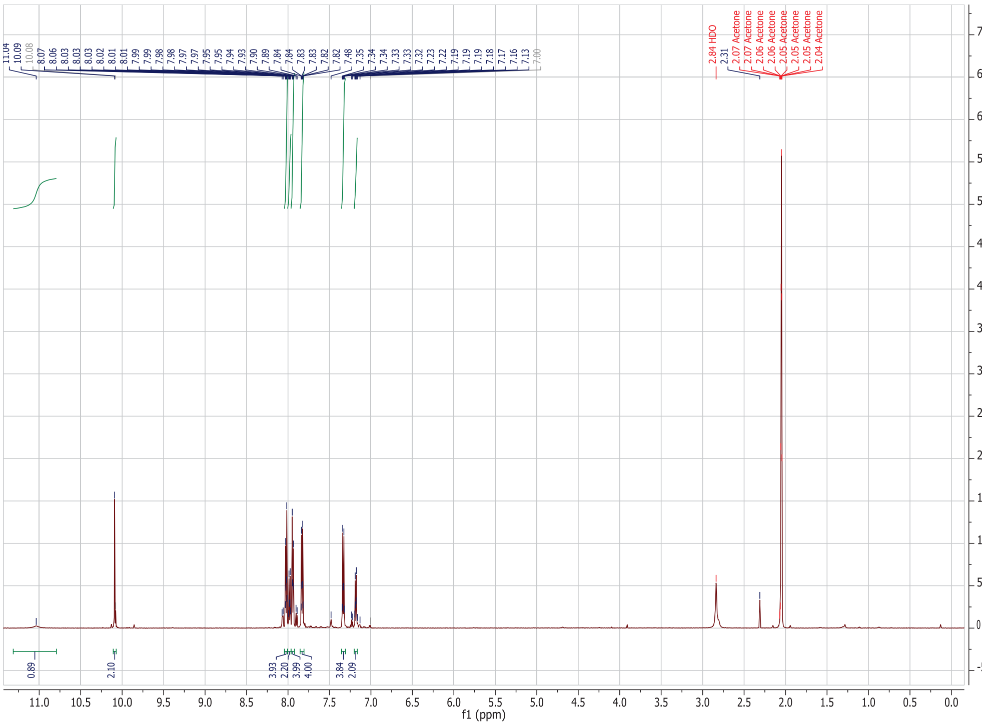

Figure S5 ^{1}H-NMR spectrum of compound **3** (4-(bis(4'-formylbiphenyl-4-yl)amino)benzoic acid) (600 MHz, acetone-d_6).

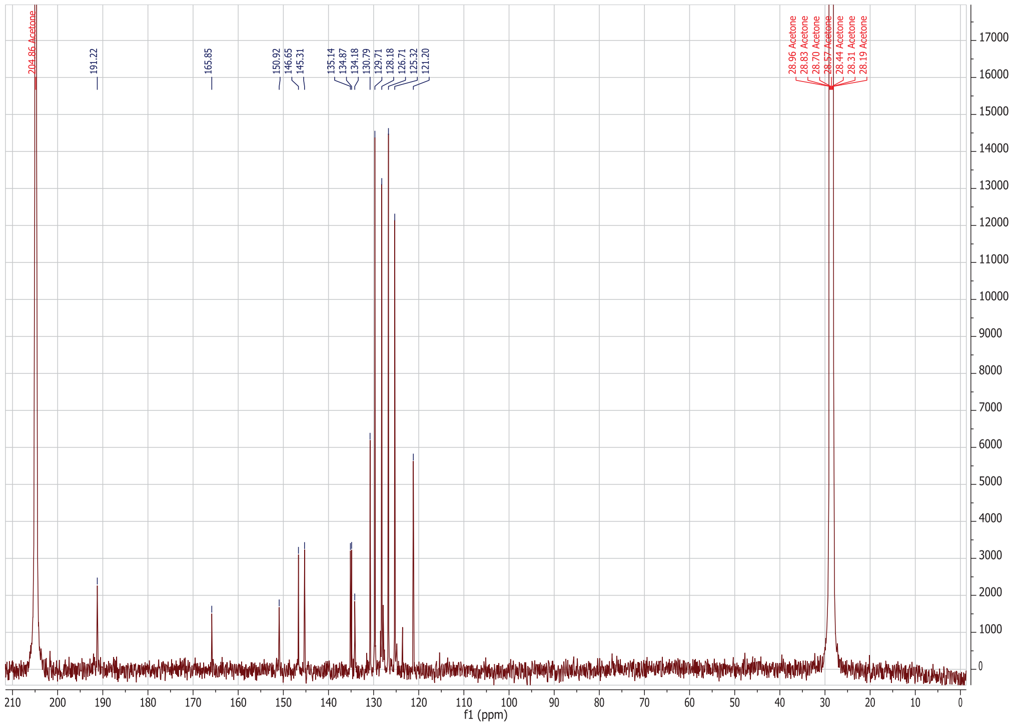

Figure S6 ^{13}C-NMR spectrum of compound **3** (4-(bis(4'-formylbiphenyl-4-yl)amino)benzoic acid) (151 MHz, acetone-d_6).

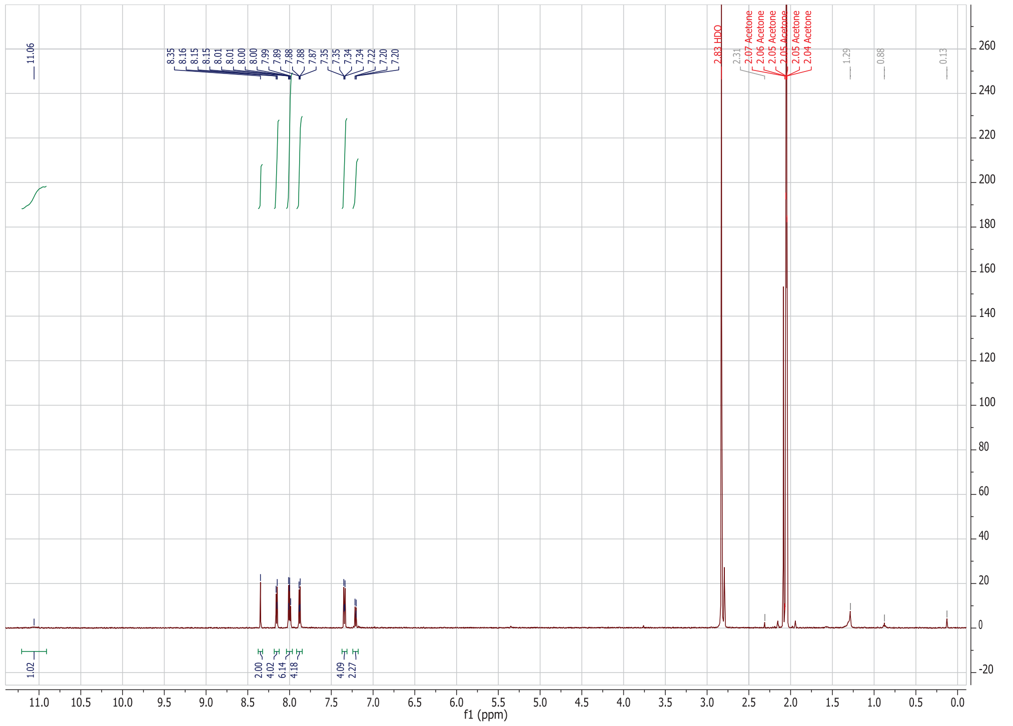

Figure S7 ^{1}H-NMR spectrum of compound **dye P4** (4-{bis[4'-(2,2-dicyanovinyl)-[1,1'-biphenyl]-4-yl]amino}benzoic acid) (600 MHz, acetone-d_6).

S2 IR spectra

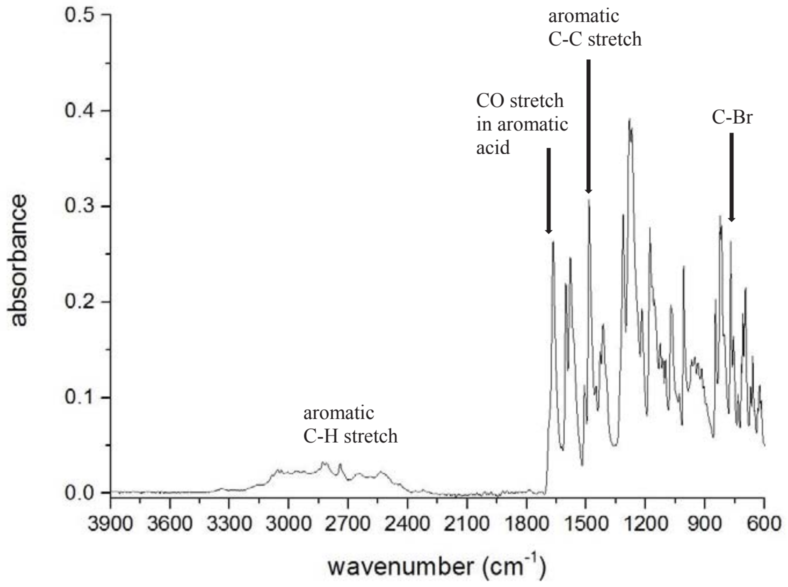

Figure S8 ATR-IR spectrum of compound **2** (4-(bis(4-bromophenyl)amino)benzoic acid, dye precursor).

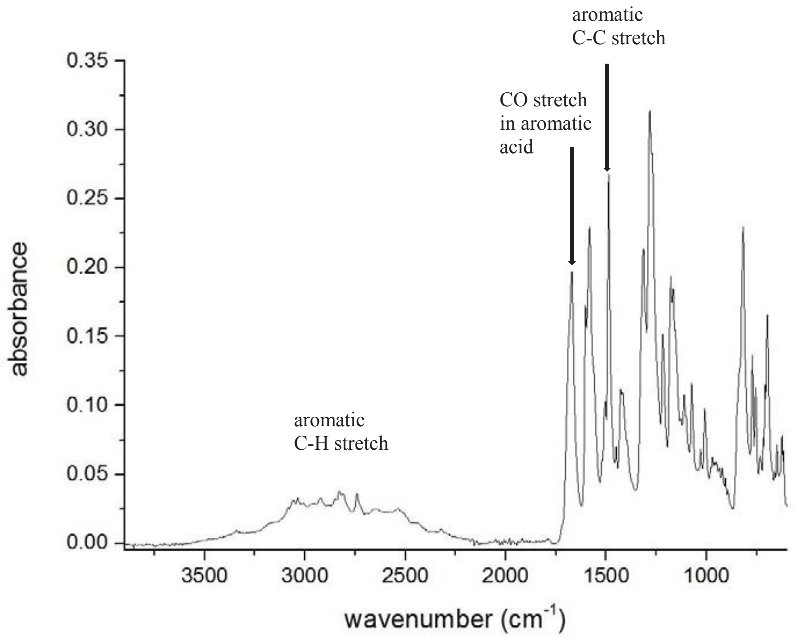

Figure S9 ATR-IR spectrum of compound **3** (4-(bis(4'-formylbiphenyl-4-yl)amino)benzoic acid).

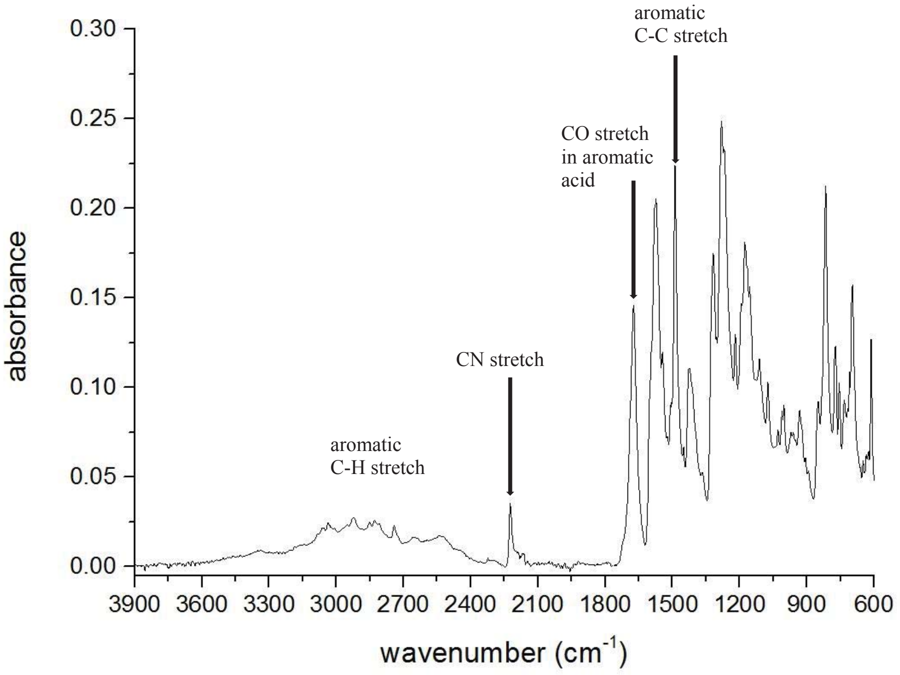

Figure S10 ATR-IR spectrum of **dye P4** (4-{bis[4‘-(2,2-dicyanovinyl)-[1,1‘-biphenyl]-4-yl]amino}benzoic acid).

S3 UV/Vis spectra

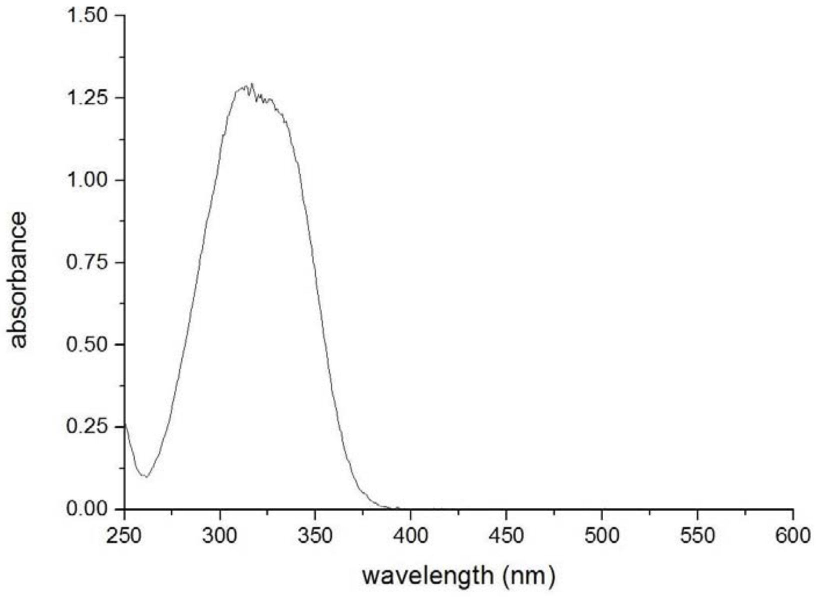

Figure S11 UV/Vis spectrum of compound **2** (4-(bis(4-bromophenyl)amino)benzoic acid, dye precursor) in acetonitrile, concentration: $5.12 \cdot 10^{-5}$ mol l^{-1}, d = 1 cm.

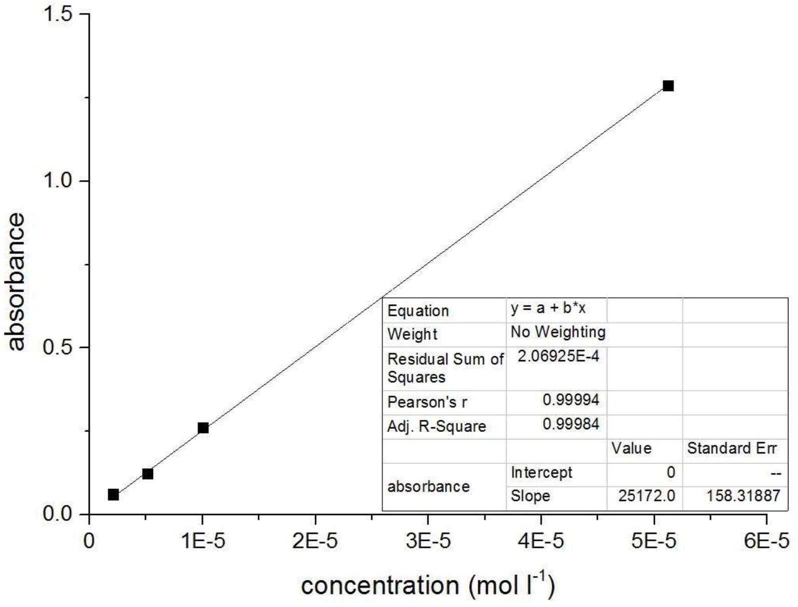

Figure S12 UV/Vis analysis of compound **2** (4-(bis(4-bromophenyl)amino)benzoic acid, dye precursor) in acetonitrile. Extinction coefficient was determined from the slope of the linear fit: $\varepsilon_{313.5,\ acetonitrile}$ = 25200 l mol^{-1} cm^{-1}.

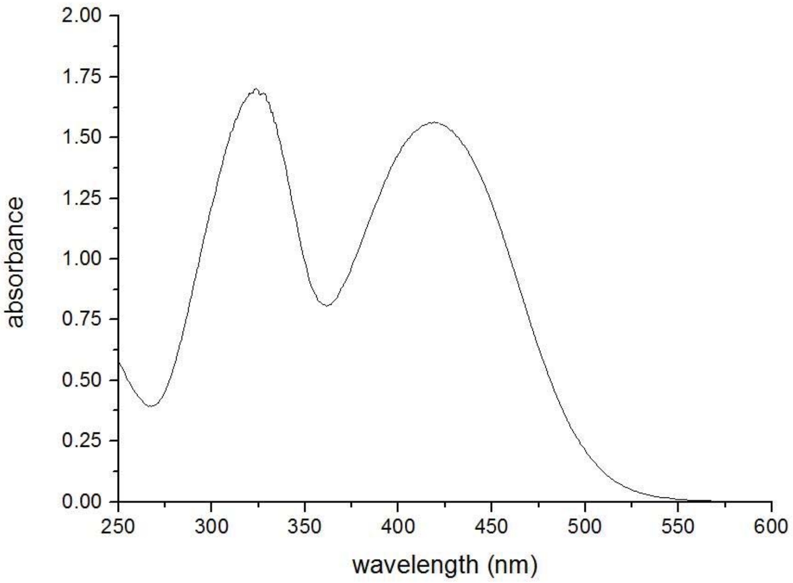

Figure S13 UV/Vis spectrum of **dye P4** (4-{bis[4‘-(2,2-dicyanovinyl)-[1,1‘-biphenyl]-4-yl]amino}benzoic acid) in acetonitrile, concentration: 4.0 · 10^{-5} mol l^{-1}, d = 1 cm.

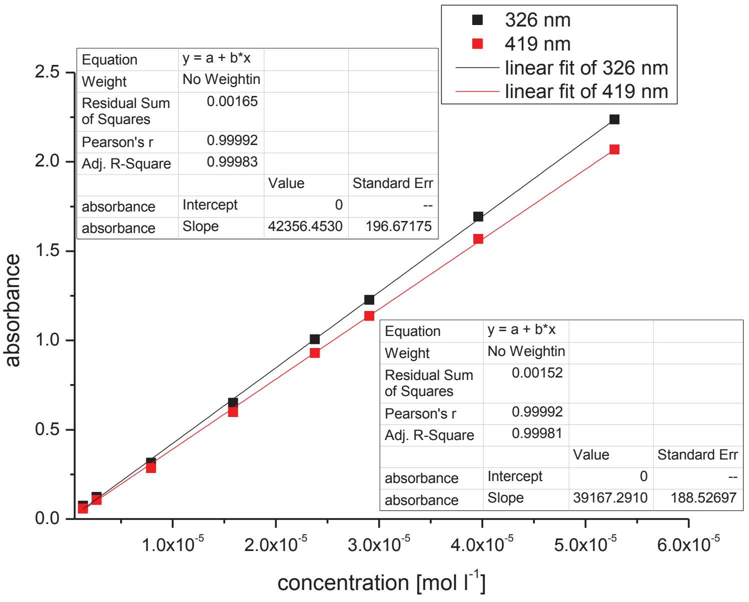

Figure S14 UV/Vis analysis of **dye P4** (4-{bis[4‘-(2,2-dicyanovinyl)-[1,1‘-biphenyl]-4-yl]amino}benzoic acid) in acetonitrile. Extinction coefficients were determined from the slopes of the linear fits: $\varepsilon_{325.5,\ acetonitrile}$ = 42400 l mol^{-1} cm^{-1}; $\varepsilon_{418.5,\ acetonitrile}$ = 39200 l mol^{-1} cm^{-1}.

S4 MS spectra

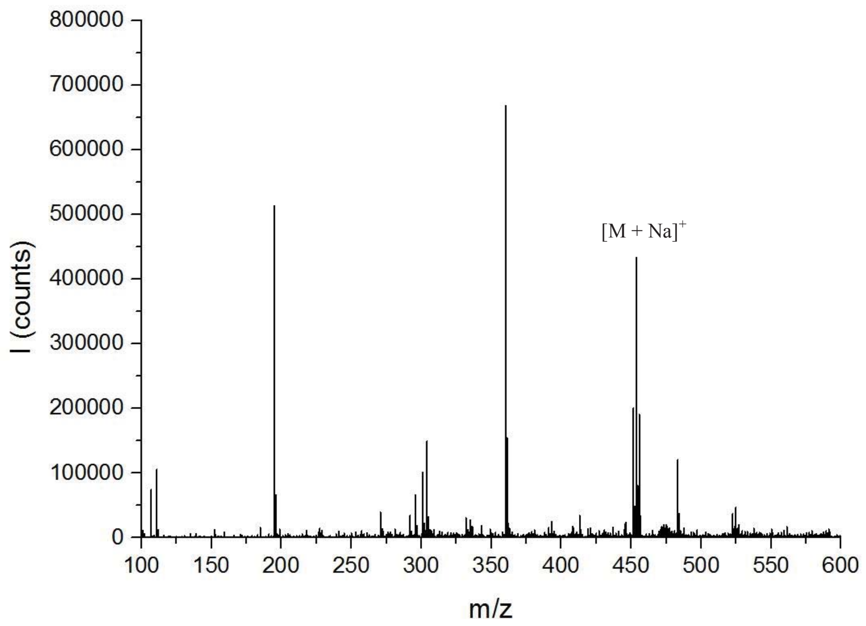

Figure S15 MS spectrum of compound **1** (4-(bis(4-bromophenyl)amino)benzaldehyde), ESI pos. mode.

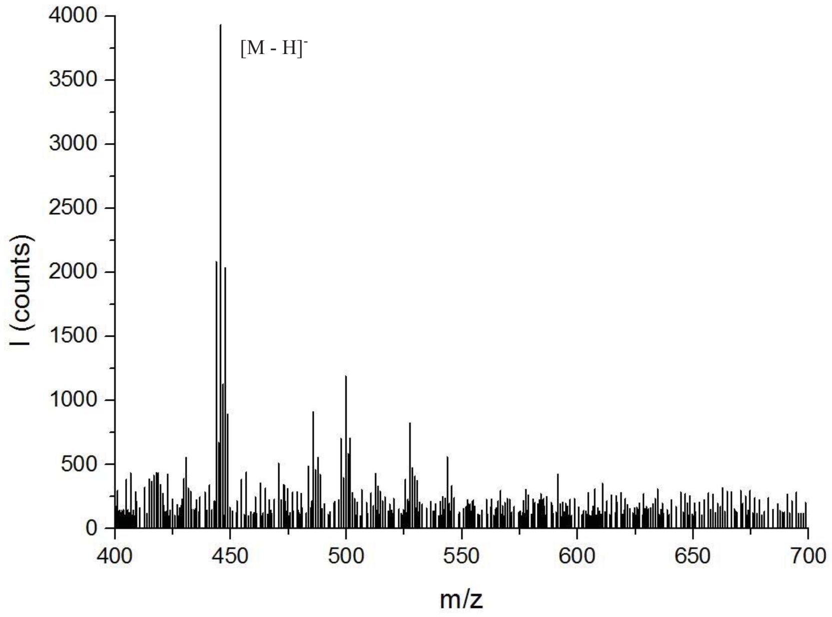

Figure S16 MS spectrum of compound **2** (4-(bis(4-bromophenyl)amino)benzoic acid), ESI neg. mode.

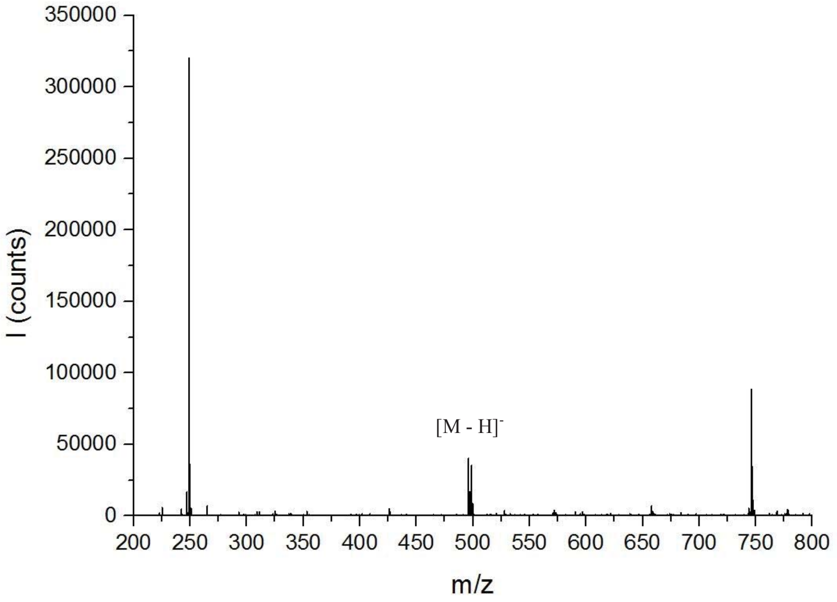

Figure S17 MS spectrum of compound **3** (4-(bis(4'-formylbiphenyl-4-yl)amino)benzoic acid), ESI neg. mode.

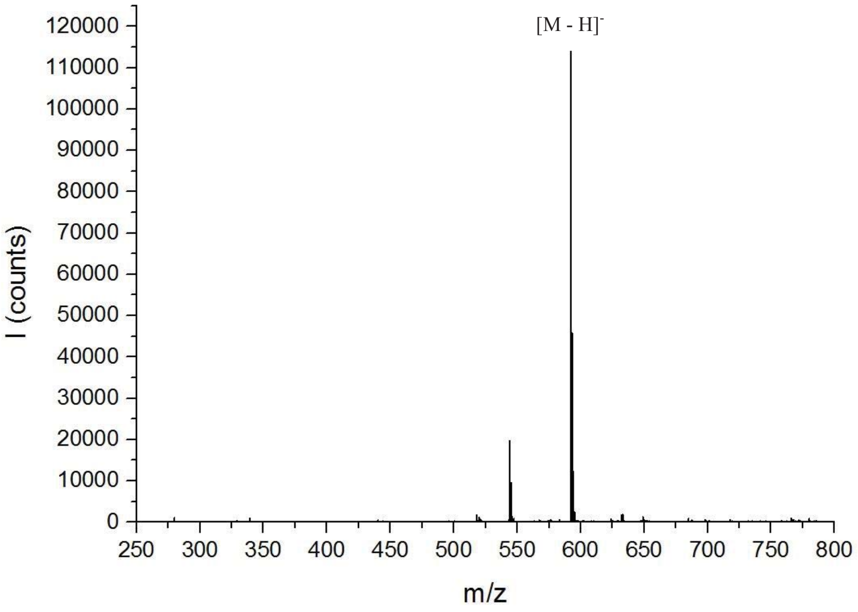

Figure S18 MS spectrum of **dye P4** (4-{bis[4'-(2,2-dicyanovinyl)-[1,1'-biphenyl]-4-yl]amino}benzoic acid), ESI neg. mode.

S5 X-Ray crystallographic details

Table S1 Crystallographic data for compound **2** (4-(bis(4-bromophenyl)amino)benzoic acid, dye precursor).

Empirical Formula	$C_{19}H_{13}Br_2NO_2$
Crystal system	Monoclinic
Space group	$P\ 2_1/c$
a/pm	2119.0(5)
b/pm	986.6(5)
c/pm	1915.5(5)
α/°	90
β/°	116.249(5)
γ/°	90
Cell volume/10^6 pm	3.59(1)
Z	8
R_1 (observed)	0.0583
wR_2 (all)	0.1344
